# The EBM-DPSER Conceptual Model: Integrating Ecosystem Services into the DPSIR Framework

**DOI:** 10.1371/journal.pone.0070766

**Published:** 2013-08-12

**Authors:** Christopher R. Kelble, Dave K. Loomis, Susan Lovelace, William K. Nuttle, Peter B. Ortner, Pamela Fletcher, Geoffrey S. Cook, Jerry J. Lorenz, Joseph N. Boyer

**Affiliations:** 1 Ocean Chemistry Division, Atlantic Oceanographic & Meteorological Laboratory, National Oceanic & Atmospheric Administration, Miami, Florida, United States of America; 2 Institute for Coastal Science and Policy, East Carolina University, Greenville, North Carolina, United States of America; 3 JHT Incorporated, Hollings Marine Laboratory, National Centers for Coastal Ocean Science, National Oceanic and Atmospheric Administration, Charleston, South Carolina, United States of America; 4 Eco-Hydrology, Ottawa, Canada; 5 Cooperative Institute for Marine and Atmospheric Studies, Rosenstiel School of Marine and Atmospheric Science, University of Miami, Miami, Florida, United States of America; 6 University of Florida Sea Grant, Gainesville, Florida, United States of America; 7 Tavernier Science Center, Audubon Florida, Tavernier, Florida, United States of America; 8 Center for the Environment, Plymouth State University, Plymouth, New Hampshire, United States of America; University of Sydney, Australia

## Abstract

There is a pressing need to integrate biophysical and human dimensions science to better inform holistic ecosystem management supporting the transition from single species or single-sector management to multi-sector ecosystem-based management. Ecosystem-based management should focus upon ecosystem services, since they reflect societal goals, values, desires, and benefits. The inclusion of ecosystem services into holistic management strategies improves management by better capturing the diversity of positive and negative human-natural interactions and making explicit the benefits to society. To facilitate this inclusion, we propose a conceptual model that merges the broadly applied Driver, Pressure, State, Impact, and Response (DPSIR) conceptual model with ecosystem services yielding a Driver, Pressure, State, Ecosystem service, and Response (EBM-DPSER) conceptual model. The impact module in traditional DPSIR models focuses attention upon negative anthropomorphic impacts on the ecosystem; by replacing impacts with ecosystem services the EBM-DPSER model incorporates not only negative, but also positive changes in the ecosystem. Responses occur as a result of changes in ecosystem services and include *inter alia* management actions directed at proactively altering human population or individual behavior and infrastructure to meet societal goals. The EBM-DPSER conceptual model was applied to the Florida Keys and Dry Tortugas marine ecosystem as a case study to illustrate how it can inform management decisions. This case study captures our system-level understanding and results in a more holistic representation of ecosystem and human society interactions, thus improving our ability to identify trade-offs. The EBM-DPSER model should be a useful operational tool for implementing EBM, in that it fully integrates our knowledge of all ecosystem components while focusing management attention upon those aspects of the ecosystem most important to human society and does so within a framework already familiar to resource managers.

## Introduction

### Ecosystem Based Management

The concept of ecosystem based management (EBM) was developed to improve resource management efficacy by applying a holistic approach that accounts for ecosystem complexity and integration rather than managing for individual issues or sectors (including individual species) [1]. EBM recognizes that: 1) the biophysical and human components of an ecosystem interact in many complex ways, 2) society relies upon and benefits from the ecosystem through ecosystem services, and 3) ecosystem services are directly and indirectly affected by multiple human activities/uses [2]. The goal of EBM is to maximize and sustain the production of ecosystem services, thus shifting management's focus from short-term economic gains or purely environmental protection/restoration towards assuring the long-term ability of an ecosystem to yield a broad suite of services important to human well-being [3].

Most management of the marine ecosystem focuses on single species, single uses, or single sectors (e.g. toxins, nutrients, development). These single-issue management approaches focus upon avoiding major damage to the ecosystem due only to the pressures attributable to that sector, or to perhaps maximize short-term single-issue outcomes (e.g., economic profitability, historic preservation, etc.). Even as a collective whole they cannot provide sufficient understanding of the tradeoffs that unavoidably occur among sectors and the cumulative effect of the different pressures being placed on the ecosystem from all of the sectors. Although EBM has been widely hailed as an improvement upon the single-sector management paradigm [4], there remain few examples of EBM being successfully implemented [5]. This is partially due to the difficulty of bridging traditional management, disciplinary, and professional boundaries [1].

In the United States, EBM was mandated in the National Ocean Policy of 2010 [6]. To successfully fulfill this mandate, we must address several inherent challenges: 1) how to integrate diverse scientific disciplines with different methodologies and approaches [7], 2) how to quantify cumulative impacts on ecosystem services [2], 3) how to develop and articulate appropriate targets that provide sustainable ecosystem services to meet human society's needs, and 4) how to incorporate and communicate uncertainty in our scientific understanding [3]; not to mention developing the operational tools required to effectively implement EBM [8]. Some of these challenges are addressed by conducting Integrated Ecosystem Assessments (IEAs); an emerging approach to synthesize and analyze existing scientific information to guide EBM development [9]. IEAs include scoping, indicator selection, and risk analysis; all of which must be informed by a synthesis of our integrated scientific knowledge about the human and biophysical components of the ecosystem [10].

### Conceptual Models to Inform Management

Integrating relevant existing knowledge is the foundation of EBM. Conceptual ecosystem models (CEMs) integrate and synthesize scientific knowledge in a manner familiar to managers and policymakers [11], [12]. If formulated properly, a CEM can address some of the challenges listed above by 1) integrating across scientific disciplines, 2) qualitatively identifying cumulative pressures, and 3) representing the scientific consensus as to how an ecosystem functions, including how humans interact with all other ecosystem components. CEMs have also been used to identify potential ecosystem indicators that can assess management success [12]–[14].

**Figure 1:**
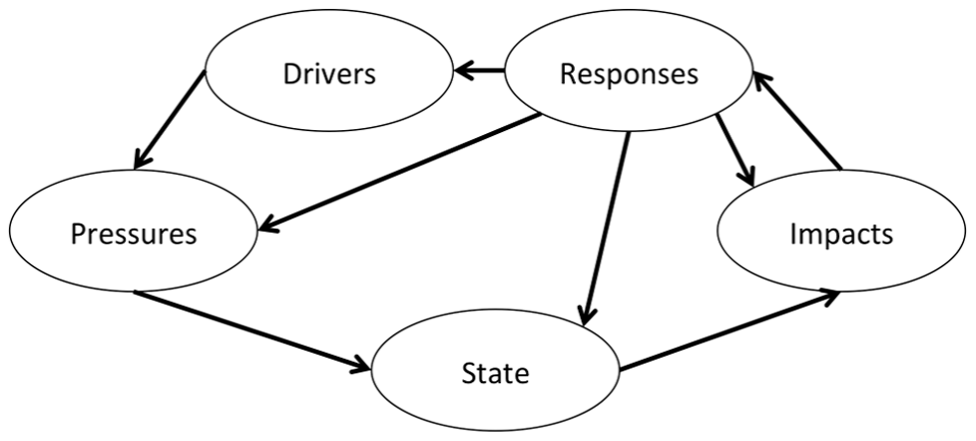
The DPSIR model. This is the DPSIR conceptual model that has conventionally been utilized for ecosystem management, assessment, indicator selection, and communication.

Initial CEMs employed to synthesize science in support of decision-making relied upon a Pressure-State-Response model [15]. The Pressure-State-Response model implicitly incorporated human society into the pressures impacting the ecosystem state and the responses that feedback to pressures. This framework evolved into the Driver, Pressure, State, Impact, and Response (DPSIR) model (Figure 1) that more explicitly depicted how human society affects ecosystem state [10], [14]. The initial impetus for the DPSIR model was to illustrate and communicate cause-and-effect relationships among indicators [16]. It has found broad application in environmental assessments of terrestrial and aquatic ecosystems due to its ability to improve communication between policymakers, stakeholders, and scientists facilitating collaborative model development [14], [17]–[19]. Because DPSIR links scientific findings with “real world” issues, it has contributed to making resource management decisions science-based [18], [20]. However, the current DPSIR model does not explicitly include ecosystem services or the values humans place on services from the ecosystem [21], and its focus upon drivers makes it difficult to fully capture the needs of local or regional human communities and less than ideal for EBM.

Drivers, which reflect underlying human needs and desires, and how they are manifest in pressures that impact the state of an ecosystem are highlighted in DPSIR CEMs. However, responses rarely directly affect drivers, but rather responses more typically directly affect pressures or the consequences of pressures by altering the methods by which humans react to or express these drivers [22]–[24]. For example, one driver is the energy requirement of a growing human population. In many situations, this driver manifests itself by the extraction of oil and the burning of fossil fuels resulting in a suite of pressures on the ecosystem including air pollution, CO_2_ emissions, ocean acidification, and anthropogenic climate change. Responses, such as changing behaviors and energy use through altering habits; carbon credits; investment in alternative energy sources; or the increased use of hybrid or electric vehicles, mitigate the burning of fossil fuels and therefore the pressures placed upon the ecosystem, but the responses do not significantly alter the driver; the energy requirement of the global human population.

The term impact in DPSIR unavoidably implies a negative environmental consequence of human activities [14]. As Svarstad et al. [19] observed traditional applications of DPSIR represented primarily the Preservationist “discourse” and the impacts module in particular did not capture the necessary information for alternative Traditionalist, Win-Win, or Promethean discourses. A partial exception to that generalization is if analyses of economic trade-offs are done in conjunction with DPSIR models both economic losses and gains are considered [25]. Nonetheless because impacts are negative environmental effects, DPSIR analyses more typically focus upon responses to these adverse environmental impacts [19], [26] and do not facilitate proactive management to sustain and maximize ecosystem services. Proactive management implies that we actively work towards a particular standard or endpoint that we find optimal or acceptable at worst, when optimal is unobtainable. For example, we try to maintain services that meet our needs and values, such as protection from storms, recreational opportunities, clean air, clean water, carbon sequestration, etc. in the locations we desire. While many of these needs and desires are universal, there are regional differences in values and preferences. In nearly all cases, maintaining these services over extended periods involves tradeoffs and decisions. As such, there exists a need for regional management tools that move resource management from a reactive approach to a proactive approach; as an analogy, making EBM a form of preventative medicine for the planet.

### Ecosystem Services and Human Well-Being

Ecosystem services are the benefits people receive from the ecosystem. As such, they reflect societal values, goals, desires, and benefits [9], [27], [28] and contribute to human well-being. “Well-being” is used by human dimensions scientists as a measure of quality of life in many contexts and is typically broken into components related to economics, environment, basic human needs, and the subjective well-being of people. On a global level, the Millennium Ecosystem Assessment describes the following components of well-being: basic material needs, freedom, health, good social relations, and personal security [27].

A distinction is often made between basic human needs and subjective well-being. Basic human needs are things required for survival such as food, water, and shelter. Subjective well-being, on the other hand, encompasses things that may not be absolutely necessary for immediate individual survival but are important to a positive emotional and psychological sense of life, such as culture and aesthetics, and may be important to long-term societal survival. Health is important to both. The absence of acute trauma and disease is a basic need, but chronic health issues contribute to subjective well-being. Developing countries focus upon meeting basic needs, while those in which those needs are being met, strive to achieve additional levels of well-being in search of a good life [27].

Aspects of well-being including environmental attributes have been addressed in the scientific literature [29] and have become the focus of assessments such as the Canadian Index of Well-being [30] and the Organization for Economic Cooperation and Development's Better Life Index [31]. Food, recreation, and storm protection are ecosystem services that benefit people directly. Not only do they provide life's basic needs, but changes in them affect economic conditions, movement of people, regulation of climate and disease, recreation and cultural opportunities, and security. As a result, changes in these ecosystem services have a wide-ranging impact upon personal well-being [27], [32]. Effective EBM assumes that regardless of an individual's recognition of ecosystem services in their lives, these services are nonetheless reflected in well-being attributes of their communities and can be measured through indicators such as health, safety, economic security, effective governance, education, food/water, housing, access to critical services, social cohesion, social conflict and environmental use [33]. Indicators of these attributes are therefore included in EBM models to provide managers with information about social and economic conditions and how these interact with natural resources.

Because ecosystem services describe the benefits that society derives from the ecosystem, both directly and indirectly [34], they are a natural bridge between the biophysical and human dimensions sciences [35]. Bridging human dimensions and biophysical sciences provides the holistic, integrated perspective of interrelated ecosystem components necessary for applying EBM [36]. Extensive scientific effort has been devoted to develop methodologies that identify, locate, and quantify the services we value [c.f. 34,37,38,39]. Despite these efforts, there are few examples employing ecosystem services to improve decision-making indicating they have yet to truly penetrate into the realm of resource management [40]. Ecosystem services will become a staple of resource management only when practical methodologies and metrics are developed that make consideration of ecosystem services tractable for decision-makers [35].

EBM must focus upon the production of and trade-offs amongst ecosystem services when evaluating the relative merit of potential management strategies or responses. Given how important ecosystem services are to EBM, it is not enough that ecosystem services be incorporated into a CEM; they need to be the focal point and explicit in the CEM. While ecosystem services can be implicitly incorporated into the DPSIR framework [17] and alternative integrated frameworks such as the Press-Pulse Dynamics model for organizing long-term research [21], we propose herein a CEM framework that explicitly merges ecosystem services directly with DPSIR to form an EBM-DPSER conceptual model. The goal of this model is to depict how the ecosystem functions and produces the ecosystem services that benefit human well-being. By including ecosystem services instead of impacts, the EBM-DPSER model captures a greater diversity of discourses providing more comprehensive information to decision–makers than a traditional DPSIR model. Although such analyses are outside the scope of this study, the EBM-DPSER model should facilitate other ecosystem service analyses, such as economic valuation, required to conduct quantitative scenario and trade-off analyses. We apply the EBM-DPSER model to the Florida Keys and Dry Tortugas marine ecosystem as a case study demonstrating the benefits and utility of this approach.

## Methods

### EBM-DPSER Framework

The EBM-DPSER model was developed within the Marine and Estuarine Goal Setting for south Florida project (MARES) [41]. The goal of MARES was to reach a science-based consensus about the defining characteristics and fundamental regulating processes of a south Florida coastal marine ecosystem that is both sustainable and capable of providing the diverse ecosystem services upon which our society depends. To achieve this goal, it was necessary to consider regional, social, political, cultural, economic, and public health factors, in both a research and management context, along with ecological variables [7], [42]–[45].

Multiple state, federal, and regional agencies share responsibility for managing the south Florida coastal marine ecosystem, but they operate with inconsistent mandates and answer to a diverse group of citizens, organizations, agencies, and businesses with diverse concerns. The need to adopt a more integrated approach to coastal ocean management has been widely recognized for many years [45]. MARES was based on the premise that through a systematic consensus-building process, science could contribute more directly and effectively to decisions being made by various management agencies. To build scientific consensus and develop a common framework for management agencies in south Florida, the MARES project required fully integrated CEMs that synthesized our knowledge about both the human dimensions and biophysical components of it's constituent ecosystem to inform integrated, holistic management, i.e. EBM, of the overall ecosystem.

The first step in the MARES process was to convene the relevant scientific experts (both biophysical and human dimensions), stakeholders, and agency representatives in a facilitated workshop and charge them with developing an integrated conceptual ecosystem model. An initial attempt was made employing the DPSIR model (Fig. 1). However, we found the DPSIR model was not a good fit for this process, in particular, or EBM in general, because of DPSIR's inability to adequately capture the full range of interactions between humans and the environment due to its reliance upon the restrictive term “impacts” which emphasizes the negative consequences of human activities upon an ecosystem [11], [14], [18], [20], [46].

A small, but increasing number of researchers applying DPSIR have also noted the omission of ecosystem services. A Web of Science search returned 127 papers on a search for “DPSIR” and only 11 papers on a search for “DPSIR” and “Ecosystem Services.” Many of these 11 papers discussed the lack of ecosystem services within DPSIR [17], [47], [48] and two actually suggested that impacts be modified to reflect changes in ecosystem services [47], [49]. Of these two, one defined impacts as the effects of environmental degradation on ecosystem services [49]. In fact only one paper did not define impacts as the strictly negative results of environmental change [47]. At least one group has attempted to link the ecosystem services cascade proposed by Haines-Young and Potschin [50] with DPSIR by associating indicators of ecosystem services and human well-being with the impacts module [51]. It is our basic contention that merely changing the definition of the impacts module is insufficient to avoid its negative connotations given its application to problems such as water pollution, poor drinking water, eutrophication, etc. [46], [52].

**Figure 2:**
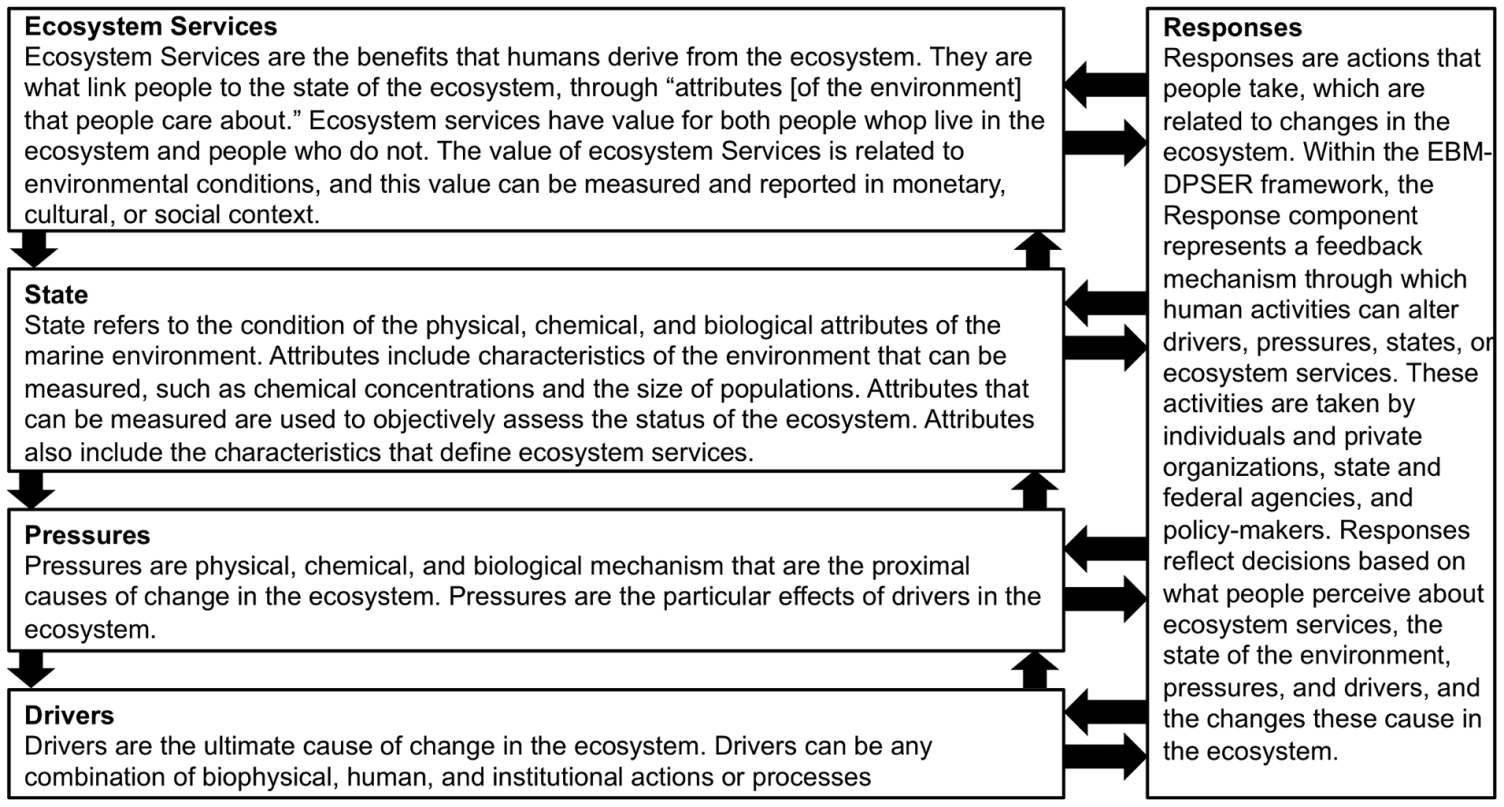
The EBM-DPSER model. The DPSIR model was modified by replacing the impacts module with ecosystem services facilitating a more complete representation of ecosystem interactions including those with human society and the associated feedbacks. Ecosystem services are at the top of the model, instead of drivers to focus attention upon the module that should be the focus of EBM decision-making.

**Figure 3:**
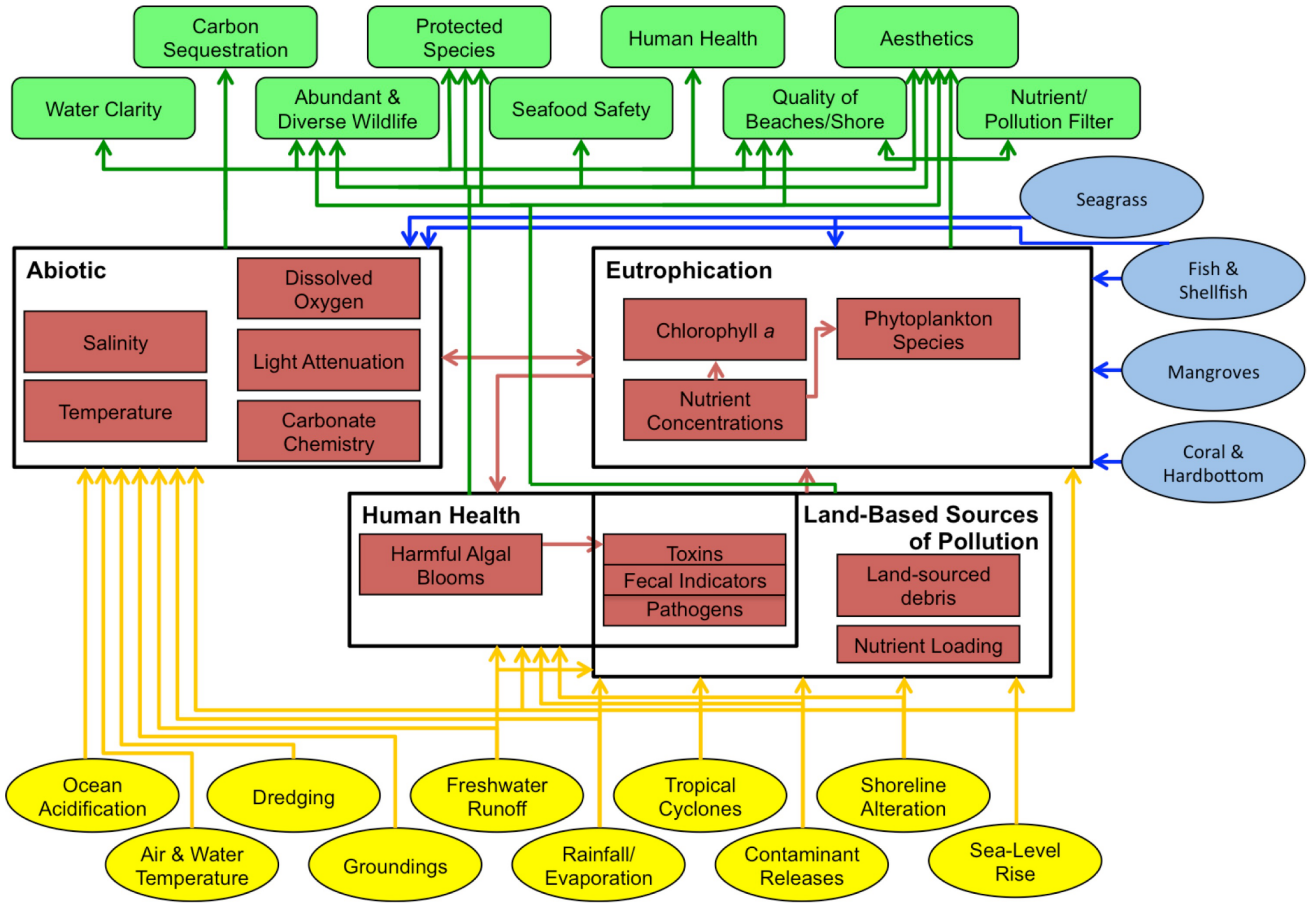
The south Florida water column sub-model. The sub-model for the water column of south Florida depicts the linkage from pressures (yellow ovals) to the state attributes that we measure (red boxes) with yellow arrows. These state attributes that we measure are organized into indicators for the water column (black outlined boxes and black text). The other states that influence the water column are depicted in the blue boxes and arrows to the right. The state attributes that we measure produce *ecosystem attributes people care about* (green boxes and arrows), which can be directly translated to ecosystem services.

To overcome this challenge and provide a more explicit definition of the relationships involved in EBM we replaced the impacts module with an ecosystem services module (Fig. 2). The exact definition of ecosystem services is still actively evolving as our scientific knowledge about ecosystem services advances [53], [54] (Table 1). Fisher et al. [54] state the need for an ecosystem services classification scheme to be determined based on the decision context. There is good reason for the definition of ecosystem services to be malleable based not just on the decision context, but also on the intended audience for your analyses and products. Our goal with EBM-DPSER was to build consensus among a broad range of scientists, managers, and stakeholders. To achieve this goal most effectively, we opted to employ the most commonly accepted definition of ecosystem services; the Millennium Ecosystem Assessment definition of ecosystem services as the benefits people obtain from ecosystems [27]. The decision context for applying the EBM-DPSER framework in MARES was to build consensus and educate decision-makers about the variety of benefits that are produced by the coastal ecosystem. Thus, ecosystem services were categorized into cultural, regulating, provisioning, and supporting services following Farber et al. [55]. In the EBM-DPSER model, state sub-models are linked to one another and as such capture supporting ecosystem services with state-to-state interactions. For example, the seagrass sub-model connects to nutrient concentrations in the water column sub-model, thus capturing the supporting service of nutrient cycling by seagrass (Fig. 3). The cultural, regulating, and provisioning ecosystem services defined by Farber et al. [55] were used as the basis for identifying ecosystem services in the EBM-DPSER conceptual model. Because the state sub-models captured supporting services, they did not need to be included explicitly within the ecosystem services module.

**Table 1:** Three common definitions of ecosystem services show significant disparity.

This Study	MEA 2005	Boyd and Banzhaf 2007	Fisher et al. 2009
the benefits people obtain from ecosystems	the benefits people obtain from ecosystems	the ecological components directly consumed or enjoyed to produce human well-being	the aspects of ecosystems utilized (actively or passively) to produce human well-being
Ecosystem Services	Ecosystem Services	Benefits and Ecosystem Services	Benefits, Intermediate and Final Ecosystem Services
Ecosystem attributes people care about		Ecosystem Services	Intermediate and Final Ecosystem Services
Ecosystem attributes people care about		Ecosystem Services	Final Ecosystem Services

The first row shows that the Millenium Ecosystem Assessment applies the broadest definition of ecosystem services with more detailed definitions provided by Boyd and Banzhaf [53] and Fisher et al [54]. In our initial EBM-DPSER model development we employed the MEA (2005) definition, because this was the most familiar definiton to the majority of participants and our goal was to build consensus. However, when the EBM-DPSER model is applied to conduct trade-off analyses of management options the other definitions for ecosystem services may be more appropriate. To help facilitate the application of other ecosystem service definitions, the table shows the linkages between the definitions of *ecosystem attributes people care about*, ecosystem services, and benefits used in this study, the Millenium Ecosystem Assessment, Boyd and Banzhaf 2007, and Fisher et al. 2009.

Sub-models were developed for each state component linking the myriad pressures to state variables that we measure to endpoints, hereafter termed *ecosystem attributes people care about*. The quantifiable state variables included in the sub-model are themselves a parsimonious subset of the descriptive characteristics that represent the overall condition of that state component [56]. The *ecosystem attributes people care about* are ecological components of the state module utilized to produce human well-being. This is the same definition of ecosystem services suggested by Fisher et al. [54]. Thus, these attributes directly correspond with ecosystem services as defined by the MEA and this project [27]. An example of these sub-models is illustrated in Figure 3, which depicts how pressures influence the attributes of the water column we measure via monitoring programs and thus affect the *ecosystem attributes people care about*.

Under the EBM guidelines, management responses should aim to provide the sustainable level of ecosystem services desired by society, making a natural link from ecosystem services to responses. Because ecosystem services play this central role within EBM, they are the key module of the EBM-DPSER model. Not only have ecosystem services been substituted into the DPSIR model, the visual representation of the EBM-DPSER model itself is inverted so ecosystem services are the pinnacle and drivers the base (Fig. 2). This de-emphasizes drivers, which are without question important; however, it is ecosystem services that relate most directly to the goals and values that motivate society to respond to changes in environmental condition.

As with more recent DPSIR applications, it is intended that the EBM-DPSER model be a causal network in the sense used by Niemeijer and de Groot [57] rather than a unidirectional chain, in order to better incorporate the multitude of complex interactions observed within an ecosystem. Figures 2 and 3 display the application of this network approach to the EBM-DPSER model and the water column element of the state module in MARES.

### Florida Keys And Dry Tortugas Case Study

**Figure 4:**
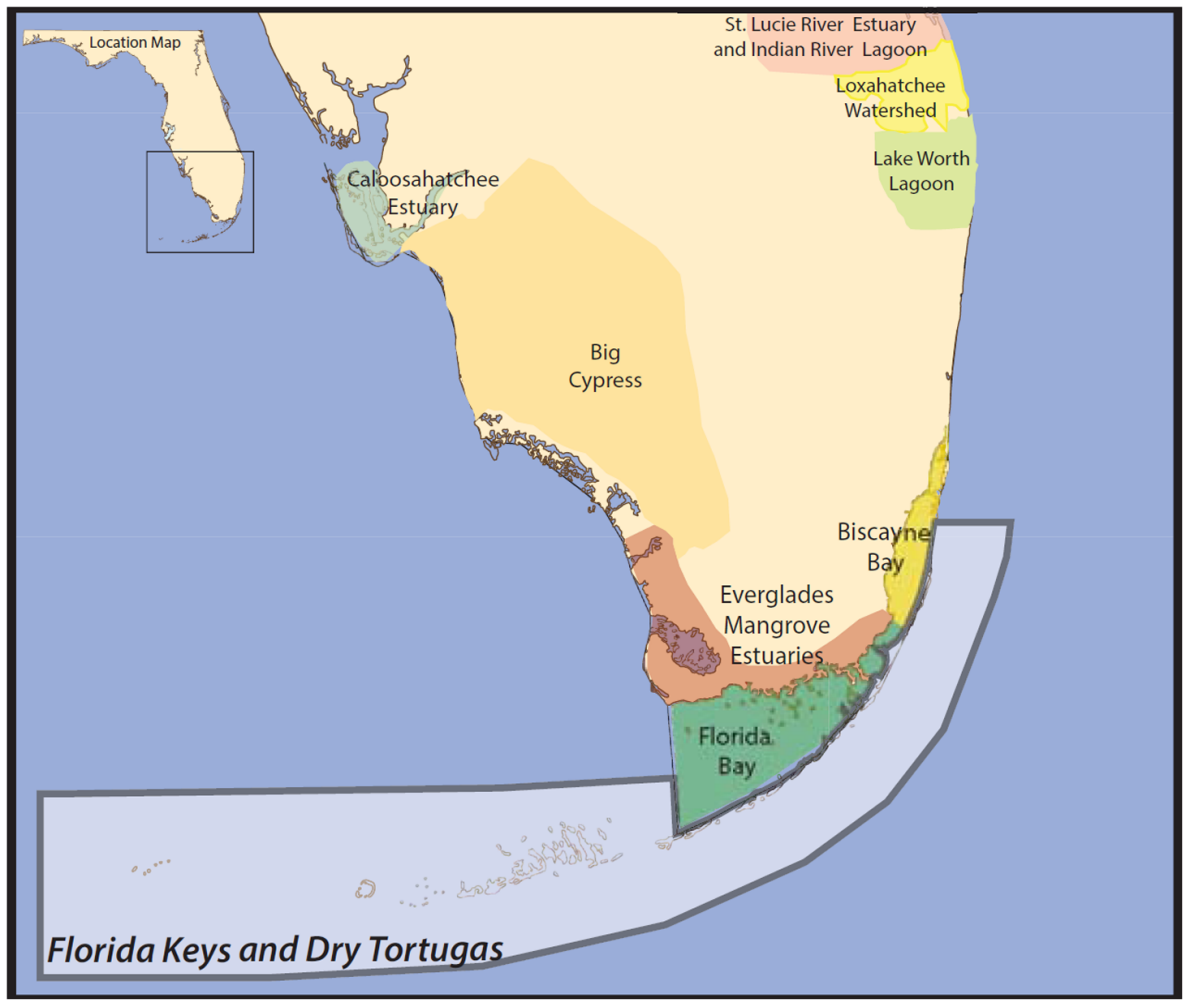
The Florida Keys and Dry Tortugas study site. The area shaded in white is the study site for the development of the Florida Keys and Dry Tortugas marine ecosystem EBM-DPSER model.

**Figure 5:**
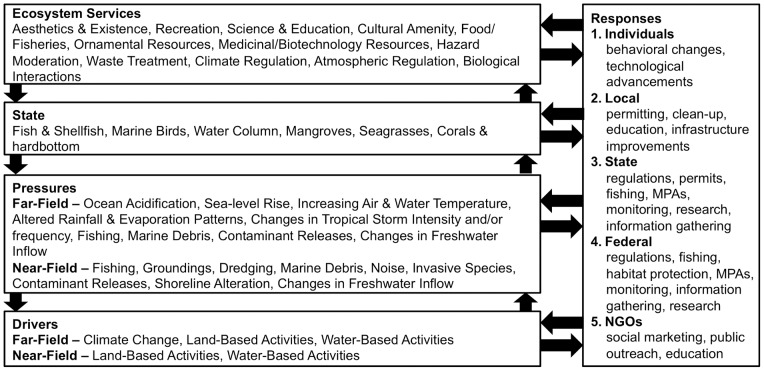
The Florida Keys and Dry Tortugas EBM-DPSER model. The EBM-DPSER model for the Florida Keys and Dry Tortugas marine ecosystem identifies the key components of each module within the CEM.

The EBM-DPSER model for the Florida Keys and Dry Tortugas (Fig. 4) was developed with input from over 60 scientists, agency resource managers, and representatives from environmental non-governmental organizations (NGOs). This group of experts worked to identify the key characteristics within each module of the EBM-DPSER framework needed to effectively synthesize our scientific knowledge and our understanding of society's relationship to the environment within the Florida Keys and Dry Tortugas study area (Fig. 5). The full report detailing the Florida Keys and Dry Tortugas EBM-DPSER model and its development is available as a technical report [41].

## Results

### Florida Keys and Dry Tortugas EBM-DPSER Case Study

The cultural, regulating, and provisioning ecosystem services put forth by Farber et al. [55] were modified and the list reduced based on input from the regional group of experts to include only those services produced by the Florida Keys and Dry Tortugas (Table 2). The cultural ecosystem services furnished by the Florida Keys and Dry Tortugas marine ecosystem are aesthetics and existence, recreation, science and education, and cultural amenity. The provisioning ecosystem services furnished by the Florida Keys and Dry Tortugas marine ecosystem are food/fisheries, ornamental resources, and medicinal and biotechnology resources. The regulating ecosystem services furnished by the Florida Keys and Dry Tortugas marine ecosystem are hazard moderation, waste treatment, climate regulation, atmospheric regulation, and biological interactions. All of these ecosystem services result in benefits to society that can be evaluated and therefore used to determine the efficacy of specific responses.

**Table 2:** Ecosystem services provided by the Florida Keys marine ecosystem.

**Cultural**	*Aesthetics & Existence*	Provide aesthetic quality of aquatic and terrestrial environments (visual, olfactory, and auditory), therapeutic benefits, and pristine wilderness for future generations	*Final Ecosystem Service*
	*Recreation*	Provide a suitable environment or setting for beach activities and other marine activities such as fishing, diving, snorkeling, motor, an non-motor boating	*Benefit*
	*Science & Education*	Provide a living laboratory for formal and informal education, and scientific research	*Benefit*
	*Cultural Amenity*	Support a maritime way of life, sense of maritime tradition, spiritual experience	*Benefit*
**Provisioning**	*Food/Fisheries*	Provide safe to eat seafood	*Final Ecosystem Service*
	*Ornamental Resources*	Provide materials for jewelry, fashion, aquaria, etc.	*Final Ecosystem Service*
	*Medicinal & Biotechnology Resources*	Provide natural materials and substances for inventions and cures	*Final Ecosystem Service*
**Regulating**	*Hazard Moderation*	Moderate extreme environmental events (e.g. mitigation of waves and stormsurge during hurricanes)	*Final Ecosystem Service*
	*Waste Treatment*	Retain storm water; remove nutrients, contaminants, and sediment from water; and dampen noise	*Final Ecosystem Service*
	*Climate Regulation*	Moderate temperature and influence or control other processes such as wind, precipitation, and evaportation	*Final Ecosystem Service*
	*Atmospheric Regulation*	Exchange CO_2_, O_2_, mercury, etc. with the atmosphere	*Final Ecosystem Service*
	*Biological Interactions*	Regulate species interactions to maintain beneficial functions such as seed dispersal, pest/invasive control, herbivory, etc.	*Intermediate Ecosystem Service*

The last column identifies these services as benefits, final or intermediate ecosystem services according to Fisher et al. 2009 [54].

In the Florida Keys and Dry Tortugas marine ecosystem EBM-DPSER model, the ecosystem state was delineated into 6 sub-models. These were the water column, fish and shellfish, and marine birds and the three dominant habitat types: coral and hardbottom, seagrass, and mangroves. Each of these sub-models, includes the linkages from pressures to *ecosystem attributes people care about* (c.f. Fig. 3). For example, in the water column ten pressures (ocean acidification, air and water temperature, dredging, groundings, changes in freshwater runoff, altered rainfall and evaporation, changes in tropical cyclone intensity and/or frequency, contaminant releases, shoreline alteration, and sea-level rise) affect the key attributes that we measure. These key attributes are grouped by the four water column indicators: eutrophication, human health, abiotic, and land-based sources of pollution. In addition, the other sub-models that influence the water column (seagrass, fish and shellfish, mangroves, and coral and hardbottom) are shown with their connections to water column attributes. The state of the water column includes nine *ecosystem attributes people care about*: water clarity, carbon sequestration, abundant and diverse wildlife, protected species, seafood safety, human health, quality of beaches and shorelines, aesthetics, and nutrient/pollution filter. In turn, these attributes directly affect the ecosystem services: aesthetics and existence, recreation, science and education, food/fisheries, waste treatment, and biological interactions.

There were 3 primary drivers linked to 14 primary pressures (Fig. 5). Drivers and pressures were organized to remain consistent with the mandates of major resource management agencies in the Florida Keys and Dry Tortugas, which have primary responsibility for either terrestrial or marine issues. The three primary drivers are climate change, land-based human activities and water-based human activities. Climate change is exclusively a far-field driver; whereas, land-based and water-based human activities produce both near-field and far-field pressures on the ecosystem. Far-field drivers and pressures are defined as those originating outside of the study area, while near-field drivers and pressures originate within the study area. Thus, the near-field drivers and pressures can be manipulated by responses that occur within the study site; whereas, far-field drivers and pressures can only be effectively managed with responses spanning a broader spatial scale, including areas outside of the study site. This organization of drivers and pressures was done to help responsible management agencies easily identify within-system responses they could employ to move the ecosystem towards a desired state.

Climate change produces five far-field pressures on the Florida Keys and Dry Tortugas ecosystem (ocean acidification, sea-level rise, increasing water and air temperatures, altered regional rainfall & evaporation, and changes in storm intensity, duration, and/or frequency). Water-based activities, in both the near- and far-field, result in fishing, marine debris, and contaminant releases. In addition, near-field water-based activities result in groundings, dredging, noise, and invasive species. Land-based activities, in both the near- and far-field, cause contaminant releases and changes in freshwater inflow. In addition, near-field land-based activities alter the adjacent shoreline.

The response module was defined in the EBM-DPSER model as actions taken as a result of the ecosystem condition. Responses occur to move the ecosystem toward a desired state or as a result of the current state of the ecosystem. For example, management regulations may be implemented that are intended to move the ecosystem to a desired state or scuba diving tourists may visit the Keys less frequently if they perceive the ecosystem to be in a degraded less desirable state. Responses occur across a range of human scales from the individual via behavioral changes to the global community via international treaties. Within the Florida Keys and Dry Tortugas these responses come from individuals, NGOs, and local, state, and federal agencies. Some identified responses include behavioral changes, technological advancements, education, outreach, social marketing, research, monitoring, infrastructure improvements, and possible regulations by responsible agencies.

Three especially significant responses were taken over the past two decades in an attempt to move the Florida Keys and Dry Tortugas marine ecosystem towards a more desirable condition. These responses were: 1) the creation of the Florida Keys National Marine Sanctuary, 2) the implementation of a rate of growth ordinance in the Florida Keys, and 3) passage of state laws requiring improvements to wastewater treatment throughout the Keys. These responses spanned various governmental levels from the Monroe County rate of growth ordinance that restricted building permits to federal government creation of the Florida Keys National Marine Sanctuary.

### Application of EBM-DPSER

To demonstrate the utility and versatility of the EBM-DPSER model, the Florida Keys and Dry Tortugas model was applied to two distinct issues relevant to EBM of the Florida Keys. First to examine the holistic ecosystem effects of a specific pressure and second to attempt to improve a specific ecosystem service in a hypothetical situation.

Growth of the human population in the Florida Keys has increased the quantity of wastewater that must be treated. Historically, septic tanks or cesspools were used, especially in individual residences. However, the presence of porous limestone throughout the Florida Keys permits seepage from these pits resulting in contaminant releases into canals and inshore waters.

Contaminant releases from wastewater carry nutrients and microbes that can have detrimental impacts on the state of the nearshore environment [58], [59]. Nutrients cause phytoplankton blooms that decrease water clarity and decay causing hypoxia in nutrient-enriched canals [59]. Nutrients can also cause macroalgal overgrowth of seagrasses and corals leading to less desirable habitats [58], [60]. The microbes released may create health problems for both humans and marine organisms, such as corals.

These impacts on the state of the nearshore environment decrease the quantity and quality of some of the ecosystem services it provides. Phytoplankton blooms decrease water clarity impacting the quality of recreation, such as snorkeling and sight fishing. Hypoxia can enrich the sediments and bottom-water of stratified canals with sulfur. When wind events overturn the canal waters, this results in an unpleasant odor decreasing aesthetics and recreational potential. Replacement of seagrass and coral with macroalgae significantly degrades the quality of marine recreation for divers and snorkelers, reduces habitat quality for fish and other wildlife, and affects pollution treatment by altering ecosystem nutrient cycling rates. The release of the wastewater-associated microbial community can cause health impacts in humans making some areas of the marine ecosystem unusable. Thus, the use of septic tanks and cesspits, decreased the ecosystem services recreation, aesthetics and existence, food and fisheries, waste treatment, and biological interactions.

Threatened and observed degradation in ecosystem services resulted in a response by the EPA, which encouraged Monroe County to reduce reliance on cesspits and septic fields by providing municipal wastewater treatment. A state law was then passed requiring advanced wastewater treatment and disposal in the Florida Keys. Analyzing this response in the EBM-DPSER context one can see it minimized the pressures placed on the ecosystem by cesspits and septic tanks, but doing so involved a trade-off; the cost of upgrading all septic tanks and cesspits to comply with the new regulations and a marked slowdown in new residential construction. The removal of septic tanks and cesspits does not require additional trade-offs with respect to ecosystem sustainability or services, because septic tanks and cesspits provide “no net benefits” to the ecosystem that are not also realized by the new advanced wastewater systems beyond the lower monetary cost to the human population.

The second application considers how one might improve the ecosystem service of recreation in the Florida Keys, specifically scuba diver recreation. This is a hypothetical scenario that would occur if it was determined that scuba diver satisfaction in the Florida Keys was less than desirable. There is some data suggesting that this is becoming an issue [61]. Ideally as is the case with this data, there will be information on the *ecosystem attributes people care about* to pinpoint the ecosystem attributes to target in response. With or without this information, the resource manager or decision-maker could apply the EBM-DPSER model to determine responses that would improve scuba diver satisfaction in the Florida Keys. Without the additional information on the *ecosystem attributes people care about*, you have to examine all the *ecosystem attributes people care about* which contribute to scuba diver recreation making the response less targeted and likely less effective. As identified through the MARES process, these attributes are water clarity, abundant and diverse marine wildlife, protected species, abundant and diverse fish, and abundant healthy coral.

For simplicity, we assume water clarity was the primary cause of scuba diver dissatisfaction similar to what was found in a satisfaction survey for the Florida Keys from 1995–1996 to 2000–2001 [61]. First, it is necessary to understand whether the conditions have actually changed over time. Leeworthy et al. [61] found that scuba divers perceived a decrease in water clarity, but systematic monitoring data did not support this perception; showing no decrease in water clarity [61]. In our fictive example we assume there was a measured decrease in water clarity.

The MARES water column sub-model has water clarity as an end-point associated with a measurable attribute (i.e. light attenuation), which itself is a function of suspended sediments, chromophoric dissolved organic matter (CDOM), and phytoplankton (Fig. 3) [62], [63]. Suspended sediment concentrations are a product of land-based sources and sediment re-suspension, a function of bottom habitat state and human activities (e.g. dredging, groundings, etc.). Land-based sources are unlikely to have a significant effect on offshore scuba diver experience, since the reefs are approximately 10km from shore. However, dredging of channels and loss of benthic habitat, especially seagrass, increases suspended sediments, negatively impacting water clarity, and indirectly impacting the abundance and diversity of fish, wildlife, and protected species. CDOM is largely land-based and thus unlikely to affect water clarity on offshore coral reefs. Algal blooms over the coral reefs would impact water clarity for scuba diving. Typically, algal blooms in the Florida Keys are caused by increased nutrient loading or a loss of grazers. Given the multiplicity of potential causes for degraded water clarity, it is necessary to determine the most significant cause(s). The need to first determine if an actual change has occurred and second determine the cause of that change to make a directed, efficient response; highlights the benefits gained from regularly collected monitoring data.

Assuming monitoring data was analyzed and it was determined that increased suspended sediment caused the degraded water clarity, the EBM-DPSER model would then be consulted to identify the pressures that affect suspended sediment concentrations. These pressures are dredging, groundings, and shoreline alteration (Fig. 3), as well as the pressures that degrade seagrass beds (groundings, dredging, contaminants releases, fishing, and changes to freshwater inflow). Responses to improve water clarity could include establishing marine protected areas that minimize damage by groundings, stabilizing sediments and promoting seagrass growth; restricting contaminant releases from vessels near the coral reefs; and ceasing dredging operations for some or all channels.

When evaluating which response or suite of responses should be implemented to improve water clarity, the EBM-DPSER model provides a useful scenario analysis tool that encapsulates the holistic effects of these responses and illuminates tradeoffs. If the management response were to ban dredging throughout the Florida Keys, this would reduce the concentration of suspended sediments in waters near channels during times dredging operations would have occurred; however, it would also restrict access for some vessels and possibly decrease the availability of deep-water refugia for some organisms, particularly during extreme temperature events. By restricting access of some vessels, there could be decreased recreational and commercial fishing opportunities and decreased tourism from cruise ships. Thus, this potential response might improve scuba diver recreation, but it would negatively impact the ecosystem services of food/fisheries, cultural amenity, ornamental resources, and possibly science & education.

## Discussion

In practice, we manage natural resources, our natural capital, for people. People are an integral participant in the ecosystem; their actions produce or reduce pressures upon the environment, but they are also the benefactors of ecosystem services. In some instances, such as wildlife refuge management or through restoration initiatives, human society chooses to enhance the environment and increase the benefits that it provides. Goals may compete, but highlighting the complex roles people play within the ecosystem should assist managers in balancing competing goals by making trade-offs explicit facilitating their efforts to balance acceptable levels of ecosystem change with protection of needed services, and provision of desired services.

By explicitly incorporating ecosystem services, use of the EBM-DPSER model facilitates and encourages ecosystem service analyses. Ecosystem services have values that can potentially be measured by human dimensions scientists. Quantitative and qualitative analytical methods can be used to produce data and tools for decision-making and to estimate the relative importance of different natural resources to particular human populations. Knowing the values that people place upon ecosystem services informs decisions that involve tradeoffs between environmental and other societal objectives and between competing objectives. Assessing the value of ecosystem services can occur within either economic or social contexts. While there is great utility in monetization for cost-benefit analyses [38], [64], [65], this must not be considered the complete valuation of the ecosystem [66]. Recreational services, as an example, are valued by society in ways that are not economic, but are still possible to quantify and interpret using other common methods [67]. Identifying the most appropriate approach whether quantitative or qualitative for the circumstances is important in order to evaluate the full range of benefits and make the most well-informed decisions [68].

Other considerations including distributive justice (the fairness associated with allocating scarce resources), sustainability, ecological stewardship, human well-being, and cultural and ethical values are important to consider in the decision-making process [69], [70]. Equity analysis (one approach to allocating scarce resources) requires estimation of the differences between groups who receive benefits and those who lose benefits under different management alternatives. There are other allocation norms associated with who does or does not receive ecosystem service benefits in the amount they want or feel they deserve, such as equality or need based allocations [71]. Sustainability and stewardship analyses focusing on the past, present, and future distributions of those services consider additional layers of complexity. Cultural and ethical considerations may place further constraints on the acceptability of different management decisions [55]. Human societies are complex with diverse perspectives on the use of ecosystem services depending on circumstances at the global-regional-local level of political or societal organization.

When conducting ecosystem service analyses it is important that ecosystem services be selected and refined based on the specific decision context [54]. Thus, if the goal is to use EBM-DPSER to examine changes in ecosystem services under different management scenarios leading to economic valuation and trade-off analyses, the definitions and classifications of ecosystem services have to be appropriate for that objective. Where those include economic valuation, it is helpful to follow either Boyd and Banzhaf [53] or Fisher et al. [54] (Table 1) and classify services as either intermediate or final. This is necessary to ensure only final services are utilized in economic valuation which avoids double-counting, a common problem in ecosystem service valuation [72].

The EBM-DPSER model is flexible and can accommodate alternative definitions and classifications of ecosystem services. The ecosystem services currently identified within the Florida Keys and Dry Tortugas case study are a mixture of intermediate and final ecosystem services, as well as benefits based upon the Fisher et al. [54] scheme (Table 2). As mentioned previously, the *ecosystem attributes people care about* which link the state to ecosystem services in the current EBM-DPSER model are defined as ecosystem services, themselves, by Boyd and Banzhaf [53] and Fisher et al. [54]. Thus, the *ecosystem attributes people care about* can and should become the ecosystem services in the EBM-DPSER model when warranted by the decision-context, such as to undertake cost-benefit analysis or to calculate green GDP.

Within the EBM-DPSER model, responses encompass human actions motivated by changes in the condition in the environment (state) or in the ecosystem services provided. Responses can affect drivers, pressures, states, or ecosystem services and represent a mechanism for anthropogenic feedback, and therefore the possibility of anthropogenic alteration. Included in responses are activities of gathering information, decision-making and program implementation that are conducted by agencies charged with making policies and implementing management actions that affect the ecosystem. The value and location of services both regulated and unregulated can have a large effect on the drivers and pressures acting on the ecosystem.

The EBM-DPSER model allows for the inclusion of the complete suite of complex positive and negative interactions between humans and their environment, thus presenting a more holistic picture of how the ecosystem, including humans, functions. This is an improvement over the antecedent DPSIR approach which primarily defines impacts as results from a degraded system and highlights societies role in causing negative impacts on the environment [49]. Capturing positive human responses that increase the production of ecosystem services or restore ecosystem health and the many positive effects of the ecosystem on human society is essential if EBM is ever to be widely implemented.

Applying scenario analysis to the DPSIR model has been recognized as a useful approach to incorporate DPSIR into management decisions [20]. This is equally true for EBM-DPSER. By applying scenario analysis to the EBM-DPSER model, the holistic effect of impending management decisions on the production of ecosystem services and ecosystem condition may be evaluated and tradeoffs among these services made explicit.

The case study and example applications in the Florida Keys and Dry Tortugas confirm the utility of applying the EBM-DPSER model to inform resource management decision-making. The EBM-DPSER model reflected a consensus as to the underlying mechanisms that create the current status of ecosystem services within the Florida Keys and Dry Tortugas; i.e., an understanding of how the *ecosystem attributes people care about* are altered by components of the ecosystem state (Figs. 3 and 5). Describing how the ecosystem states relate to ecosystem services can be useful to managers attempting to implement EBM. Identifying which ecosystem states produce a given ecosystem service and which pressures impact these states highlights potential underlying causes of ecosystem service production levels. This enables ready recognition of those responses that improve the delivery and sustainability of an ecosystem service. Placing ecosystem services into a model (DPSIR) that has been widely accepted by the management community should provide a politically and socially realistic path towards the incorporation of ecosystem service analysis into decision-making [18], [20].

As in the example of scuba diver satisfaction, once a problem is identified it will often be necessary to undertake a more detailed study that characterizes the ecosystem attributes, which are below a desired level. The information from that study will facilitate the most effective targeted response. The EBM-DPSER model may also be applied to conduct scenario analyses of alternative responses to determine their holistic effect and identify potential trade-offs. The EBM-DPSER model illustrated that a hypothetical response to ban dredging does not just decrease suspended sediment, but also has negative effects including access for vessels and providing refugia. These positive benefits provided by dredged channels increase the production of ecosystem services. By illuminating these positive effects, in addition to the negative impacts, EBM-DPSER provides a more complete identification of the tradeoffs inherent in any response. Identifying these tradeoffs between ecosystem services with respect to management goals is recognized as a key informational need for EBM [73]. Managers must consider tradeoffs. If they determine that detrimental use of the area is necessary for society and that negative impacts are unavoidable then it will be important for managers to identify alternative areas and direct recreational users and commercial fishers to them to obtain the ecosystem services that they demand.

Identifying the trade-offs qualitatively is a first step towards quantification of these trade-offs. Quantification is difficult in many cases, because of the frequent asymmetry of effects of management actions on ecosystem services. The effect of management responses are often direct and localized, while the benefits from ecosystem services are more diffuse and indirect [74]. Moreover, quantification is confounded by a lack of consistent units. One approach to overcome the lack of consistent units would be to use expert opinion to scale or ordinate these linkages following a method similar that employed by Altman et al. [2] to quantify the linkage between pressures and ecosystem services.

Progress towards quantification of interactions among pressures, states, and ecosystem services would make scenario analyses and the inherent trade-offs among scenarios quantitative rather than qualitative and better inform resource management decisions. It would also allow for holistic risk assessment following a modification of the Altman et al. approach [2] to incorporate the effect of multiple pressures upon ecosystem states and in turn upon ecosystem services. This holistic risk analysis would determine the pressures causing the largest loss of ecosystem services and the ecosystem services subject to the largest stress from the cumulative effect of all pressures.

Both scenario analyses and holistic risk assessment account for the impact of multiple human uses on ecosystem services simultaneously. Although this is an underlying assumption in EBM, there are few practical tools that account for the cumulative effect of multiple human uses on ecosystem services [2]. If we fail to account for the cumulative effect of pressures upon ecosystem services and ecosystem state, the science used to inform decision-making will be incomplete. At the same time we need to account for human societal uses to calculate tradeoffs. By coupling risk assessment and scenario analysis, the relative reduction or increase in risk to ecosystem services could be evaluated for each potential response.

The EBM-DPSER model is an important step towards effectively informing ecosystem management for the benefit of humans; whereas, the DPSIR model is designed to inform ecosystem management to protect the ecosystem from human impacts. The EBM-DPSER model directly addresses several of the inherent challenges to EBM, while maintaining a focus on EBM's fundamental objectives. It integrates diverse scientific disciplines and captures more completely the multitude of complex interactions between the biophysical and human components of the ecosystem. By highlighting ecosystem services it emphasizes the extent to which society relies upon and benefits from the ecosystem. This inclines EBM toward proactive intervention rather than strictly reactive management. The linking of pressures to states to ecosystem services permits at a minimum the qualitative assessment of the cumulative impacts of pressures upon ecosystem services and captures the direct and indirect effect of multiple human uses on ecosystem services, as well as the loss of ecosystem services to human society. This is an important first step towards quantifying these complex interactions. As shown in the example applications, the EBM-DPSER model can assist in bridging the communication gap between human dimensions scientists, biophysical scientists, and resource managers thereby providing EBM with a useful operational tool.
